# Host Lipid Rafts Play a Major Role in Binding and Endocytosis of Influenza A Virus

**DOI:** 10.3390/v10110650

**Published:** 2018-11-18

**Authors:** Dileep Kumar Verma, Dinesh Gupta, Sunil Kumar Lal

**Affiliations:** 1 International Centre for Genetic Engineering and Biotechnology, Aruna Asaf Ali Marg, New Delhi 110067, India; dileep21feb@gmail.com (D.K.V.); dinesh@icgeb.res.in (D.G.); 2 School of Science and Tropical Medicine & Biology Platform, Monash University, Malaysia, Bandar Sunway, Selangor, DE 47500, Malaysia

**Keywords:** lipid raft, Cholesterol, Methyl-β-Cyclodextrin, Ganglioside GM1, Raft-dependent endocytosis

## Abstract

Influenza still remains one of the most challenging diseases, posing a significant threat to public health. Host lipid rafts play a critical role in influenza A virus (IAV) assembly and budding, however, their role in polyvalent IAV host binding and endocytosis had remained elusive until now. In the present study, we observed co-localization of IAV with a lipid raft marker ganglioside, GM1, on the host surface. Further, we isolated the lipid raft micro-domains from IAV infected cells and detected IAV protein in the raft fraction. Finally, raft disruption using Methyl-β-Cyclodextrin revealed significant reduction in IAV host binding, suggesting utilization of host rafts for polyvalent binding on the host cell surface. In addition to this, cyclodextrin mediated inhibition of raft-dependent endocytosis showed significantly reduced IAV internalization. Interestingly, exposure of cells to cyclodextrin two hours post-IAV binding showed no such reduction in IAV entry, indicating use of raft-dependent endocytosis for host entry. In summary, this study demonstrates that host lipid rafts are selected by IAV as a host attachment factors for multivalent binding, and IAV utilizes these micro-domains to exploit raft-dependent endocytosis for host internalization, a virus entry route previously unknown for IAV.

## 1. Introduction

Flu caused by influenza A virus (IAV) affects millions of people by seasonal and rare pandemic outbreaks [1]. IAV belongs to the *Orthomyxoviridae* family of viruses containing a negative-sense, single-stranded, segmented RNA genome distributed along eight segments encoding 11 major viral and some accessory proteins. Each virus is structurally divided into three major components: a central core of RNA genome (vRNA) with associated proteins surrounded by matrix proteins (M1) and the outermost host derived lipid bilayer with two key viral proteins: hemagglutinin (HA) and neuraminidase (NA) [2].

The first encounter between the virus and the target host cell occurs at the plasma membrane (PM). Lipid rafts are organized subdomains within the PM and are rich in glycosphingolipids and cholesterol [3]. Rafts are known to perform critical functions in a variety of cellular processes such as membrane signaling, regulation of membrane trafficking, endocytosis, and entry of pathogens and bacterial toxins [4]. For viruses, host rafts have been reported to serve as a platform for virus attachment and entry, virion assembly, and they are a preferred site for viral budding in the case of enveloped viruses [5,6]. Clustering of host lipid rafts also induces a signaling platform and activates receptor tyrosine kinases RTKs upon multivalent binding for virus uptake [7]. Cyclodextrins are commonly used to extract cholesterol and disrupt rafts, leading to inhibition or arrest of raft associated processes including raft-dependent endocytosis [8].

The attachment of IAV to its host cell is mediated by hemagglutinin (HA), a multifunctional viral protein that binds to terminal sialic acid (SIA), the primary receptor for IAV [9]. The interaction of HA-SIA is documented to be of low affinity, therefore, IAV utilizes multiple HA-SIA interactions [10], thereby achieving high avidity on the host surface [11]. However, the mechanism for achieving this multivalent binding on the host surface still remains elusive. IAV attachment to the host cell is followed by internalization into the endosome, trafficking to the perinuclear region, and fusion of the viral-endosomal membranes leading to release of the IAV genome into the cytosol for nuclear import [2]. Thus far, the known internalization route for IAV includes clathrin dependent endocytosis (CDE), clathrin independent endocytosis (CIE), caveolin independent endocytosis, and macropinocytosis [12,13]. Lipid rafts are reported to induce endocytosis upon ligand binding, a process known as “raft-dependent endocytosis” [14,15]. Endocytosis via lipid rafts has been categorized into three different pathways, namely dynamin-dependent endocytosis of caveolae, noncaveolar vesicular carriers, and dynamin-independent endocytosis via noncaveolar tubular intermediates, and rafts are reported to be utilized by various ligands for internalization, including viruses [16]. However, it is still unclear whether IAV uses raft-dependent endocytosis for internalization and entry. Once vRNPs are inside a host nucleus, the replication and transcription begins, followed by export, assembly, and budding of progeny viruses [2].

In this study, using ganglioside 1 (GM1)-cholera toxin-based lipid raft staining and biochemical raft isolation, we show that IAV select host rafts for polyvalent host binding. Further, by applying cyclodextrin based raft disruption we also demonstrate significant reduction in IAV attachment in cholesterol-depleted cells confirming this hypothesis. Additionally, using Methyl-β-Cyclodextrin (MBCD) based inhibition of raft-dependent endocytosis; we identify raft-dependent endocytosis as an internalization route for IAV in addition to the previous known entry routes for IAV.

## 2. Methods

### 2.1. Cell Culture and Viral Strains

Human lung epithelial cells A549 were purchased from ATCC and grown and maintained in DMEM (Hyclone, Logan, UT, USA) supplemented with 10% fetal calf serum (Hyclone) and a penicillin–streptomycin solution (100 units per mL; Invitrogen, Carlsbad, CA, USA) in a 5% CO_2_-containing environment. For virus infection, influenza virus A/Aichi/2/1968 (X-31) strain was used at a multiplicity of infection (M.O.I.) of 10 unless specified otherwise.

### 2.2. Chemicals and Antibodies

MBCD and MTT (3-4,5-Dimethylthiazol-2-yl)-2,5-Diphenyltetrazolium Bromide) were purchased from Sigma-Aldrich. For raft protein isolation, a focus signal proteins kit (G-biosciences, Saint Louis, MO, USA) was purchased and used according to the manufacturer’s protocol. To visualize lipid rafts, a Vybrant Lipid Raft Labeling Kit was purchased from Molecular Probes (Thermofisher Scientific, Waltham, MA, USA). For confocal microscopy and Flow Cytometric studies, Anti-NP-FITC (anti-nucleoprotein-fluorescein isothiocyanate) conjugated antibody was purchased from Abcam (Bristol, UK). For Western blotting, anti-GAPDH (Glyceraldehyde 3-phosphate dehydrogenase) antibody (Santa Cruz Biotechnology Inc., Santa Cruz, CA, USA), anti-actin-HRP antibody (Sigma Aldrich, St. Louis, MO, USA), and anti-NP antibody (Abcam, UK) was used.

### 2.3. Lipid Raft Labeling, Disruption, and Inhibition of Raft-Dependent Endocytosis

Lipid rafts were fluorescently labeled in vivo using a Vybrant Lipid Raft Labeling Kit (molecular probes) according to the manufacturer’s protocol. To disrupt lipid rafts and raft-dependent endocytosis, A549 cells were treated with 10 mM MBCD for 1 h at 37 °C in pre-warmed serum and antibiotic free DMEM medium. After treatment, cells were washed with 1× PBS three times to ensure complete removal of MBCD and processed for viral infection and analysis. We did not use cholesterol synthesis inhibitors because of reported intracellular accumulation of GM1 [17].

### 2.4. Virus Infection

For virus infection, seeded A549 cells were first washed with 1× PBS three times followed by incubation with IAV in DMEM with 0.3% BSA (GIBCO, Waltham, MA, USA) either at 4 °C or 37 °C for a period of one hour with gentle rocking at every 15 min′ interval. After 1 hour, unbound viruses were removed by washing with 1× PBS, cells were harvested at the indicated time and subjected to experimental analysis.

### 2.5. Confocal Microscopy

To visualize lipid rafts, A549 cells were subjected to raft staining (Vybrant Lipid Raft Labeling Kit, Waltham, MA, USA) on ice, fixed, and mounted on glass slides in Prolong Gold anti-fade reagent with DAPI (4′, 6-diamidino-2-phenylindole) (Invitrogen, Carlsbad, CA, USA). To detect surface bound or internalized virus, A549 cells were incubated with IAV (X-31) at an M.O.I. of 10. After indicated incubation, cells were fixed with 4% paraformaldehyde in 1× PBS for 30 min at 4 °C or RT. Then, cells were mixed with 0.5% BSA in 1× PBST with 0.3% Triton X-100 (blocking and permeabilization) for 1 h at room temperature followed by staining with mouse anti-NP-FITC conjugated antibody (dilution 1:250 in blocking buffer) for 90 min at room temperature. After staining, cells were washed with 1× PBS (3 times, 5 min each) and mounted in Prolong Gold anti-fade reagent with DAPI (Invitrogen). The slides were visualized using Leica DM6000 CFS Confocal Microscope.

### 2.6. MTT Assay

To assess cell viability, A549 cells were seeded at the density of 1 × 10^6^ cells/well in a 6-well plate. After 24 h, cells were treated with 10 mM MBCD for 1 h, untreated cells served as control. After treatment, cells were washed with 1× PBS and 100 μL MTT was mixed with 900 μL 1× PBS into each well, followed by incubation at 37 °C for 2 h to allow the formation of formazan. The medium was then removed, washed, and 1 mL DMSO was added to each well. After gentle mixing, the absorbance was measured using an ELISA plate reader at 570 nm wavelength. Only DMSO was used as the blank (medium) control.

### 2.7. Isolation of Raft Proteins and Western Blotting

To study IAV and host raft interaction, IAV infected A549 cells were subjected to raft protein isolation using a focus signal protein isolation kit (G Biosciences, St. Louis, MO, USA) according to the manufacturer’s protocol and processed for Western blotting. For IAV host binding and internalization studies, A549 cells were either subjected to MBCD treatment (10 mM, 1 h) or left untreated (Control) and then incubated with IAV either at 4 °C or 37 °C. After the indicated time, cells were then harvested, lysed, and processed for Western blotting.

### 2.8. qRT-PCR

To quantify vRNA, IAV infected A549 cells (treated or untreated) were subjected to isolation of total cellular RNA using trizol. Subsequently, after DNase I treatment, 2 µg of isolated RNA was reverse-transcribed into cDNA using ThermoScript RT-PCR System (Invitrogen) in a volume of 20 µL using the manufacturer’s protocol. The obtained cDNA was further diluted into 1:10 fold and 5 µL of diluted cDNA was used to set up a SYBR Green based real-time PCR reaction in a volume of 25 µL, and it was analyzed using StepOnePlus^TM^ real-time PCR instrument (Thermofisher Scientific, USA). The primers used were, NP-forward primer 5′-CAG GTA CTG GGC CAT AAG GAC-3′, NP-reverse primer 5′-GCA TTG TCT CCG AAG AAA TAAG-3′, Beta-actin, forward primer 5′-ACC AAC TGG GAC GAC ATG GAG AAA-3′ and reverse primer 5′-TAG CAC AGC CTG GAT AGC AAC GTA-3′ or acidic ribosomal phosphoprotein (ARPP,) forward primer 5′-GCA CTG GAA GTC CAA CTA CTTC-3′ and ARPP reverse primer 5′-TGA GGT CCT CCT TGG TGA ACAC-3′. The relative fold changes were calculated using the ∆∆Ct method.

### 2.9. Flow Cytometry

To measure the virus infected cell population, A549 cells were seeded in 6-well plates (~10^6^ cells/well) and incubated at 37 °C in 5% CO_2_ for 24 h. The following day, these cells were either subjected to treatment with 10 mM MBCD or left untreated (Control) and then incubated with IAV either at 4 °C or at 37 °C. Next, these cells were incubated for the indicated time, harvested, washed, and suspended in 200 μL of 1× BD Cytofix/Cytoperm solution (BD Biosciences, San Jose, CA, USA) and incubated for 20 min at 4 °C for fixation and permeabilization. After that, cells were washed with 100 μL of 1× BD Perm/Wash buffer three times and viruses were stained with anti-NP-FITC antibody (dilution 1:250) in 1× BD Perm/Wash buffer. Unbound antibodies were washed away with 1× BD Perm/Wash buffer, samples were diluted at 1:5 ratios in 1× PBS and then analyzed using a fluorescence-activated cell sorting (FACS) Calibur flow cytometer (BD Biosciences) and Flowjo software.

## 3. Results

### 3.1. Influenza A Virus Bind Selectively to Host Membrane Rafts

**Figure 1:**
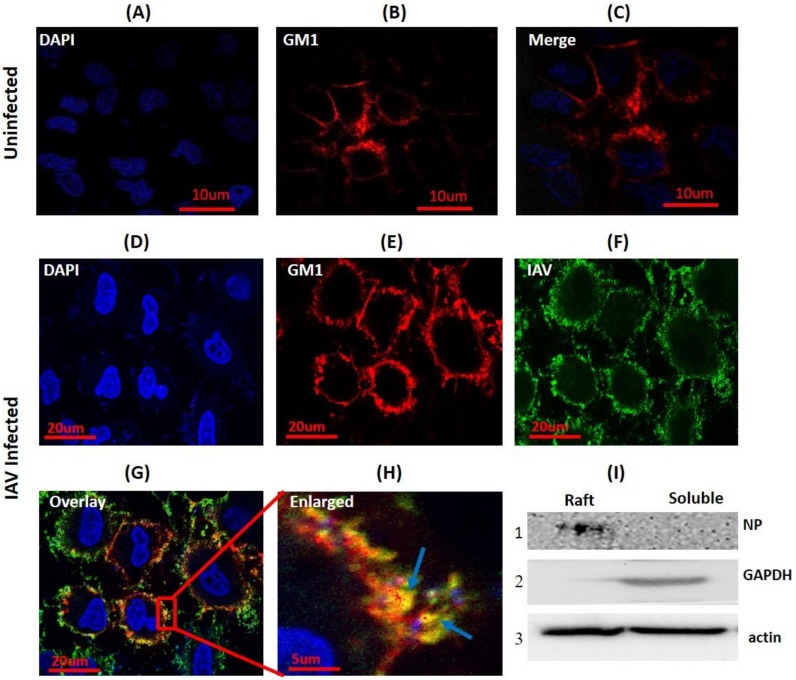
Influenza A virus (IAV) co-localizes with host membrane lipid rafts during binding. (**A**–**C**) CTB-594 stained (4 °C) A549 cells (uninfected control). Patchy distribution of ganglioside 1 (GM1) (rafts) is visible (red); blue, DAPI (4′, 6-diamidino-2-phenylindole) nuclear staining. (**D**–**G**) IAV incubated (4 °C) A549 cells stained with CTB-594 (4 °C). Surface bound viruses were stained with anti-NP-FITC (anti-nucleoprotein-fluorescein isothiocyanate) antibody (Green). Yellow color and arrows indicate IAV co-localization with GM1; blue, DAPI nuclear staining. (**H**) Enlarged view of indicated section of image G, magnification ~3X. (**I**) Western blot of raft proteins isolated from IAV incubated (4 °C) A549 cells. NP was detected in the raft fraction (I, panel 1). GAPDH (Glyceraldehyde 3-phosphate dehydrogenase) was used as cytosolic marker and beta-actin as internal control.

To investigate whether IAV utilizes the host membrane rafts during binding, seeded A549 cells were either incubated with IAV (strain A/X-31 H3N2) at 4 °C to arrest the attached virus on the host cell surface or left uninfected (mock). Then, these cells were subjected to cholera toxin-GM1 based lipid raft labeling (Red) at 4 °C to prevent internalization of GM1-Cholera Toxin-B (CTB) complex and to visualize PM rafts in the presence and absence of ligand (IAV) binding. Subsequently, cells were fixed using 4% paraformaldehyde, permebealized, and surface bound viruses were stained with anti-NP-FITC antibody (Green). When visualized under a confocal microscope, uneven, patchy, and distinct lipid rafts were clearly visible in the cell membrane from both uninfected and IAV infected cells (Figure 1B,C,E). In IAV attached cells, we observed clear co-localization of IAV with GM1 shown as a yellow color in the overlay confocal image (Figure 1G) and highlighted by arrows (Figure 1H). Also, two coefficients for co-localization viz. Pearson’s correlation coefficient (PCC = 0.71) and Mander’s overlap coefficient (MOC = 0.89) were calculated, and both coefficients supported the observed co-localization.

Next, in order to validate the interaction of host lipid raft and IAV, A549 cells were incubated with IAV at 4 °C for 1 h and subjected to biochemical raft isolation as DRM (detergent-resistant membranes) using a Focus signal protein kit. Both DRM and detergent soluble fraction were then subjected to SDS-PAGE and Western blotting. We detected viral protein (NP) in the isolated raft fraction (Figure 1I, panel 1). Presence of IAV protein in the isolated raft fraction and observed co-localization clearly indicate that host raft serves an important role as an attachment factor in IAV binding.

### 3.2. Disruption of Host Lipid Rafts Significantly Reduces IAV Host Binding

**Figure 2:**
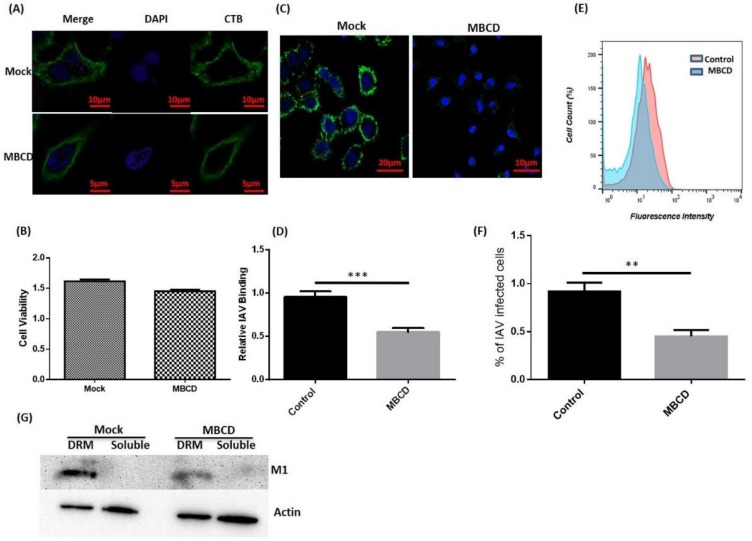
Disruption of host lipid rafts and IAV binding. (**A**) CTB-488 stained A549 cells (untreated and Methyl-β-Cyclodextrin (MBCD) treated). (**B**) Assessment of cell viability by MTT (3-4,5-Dimethylthiazol-2-yl)-2,5-Diphenyltetrazolium Bromide) assay. Error bars represent ±SD. (**C**) IAV incubated (4 °C) A549 cells, (**D**) Relative IAV host binding represented as mean fluorescence intensity (FITC). *** *p* < 0.0002, calculated by student’s *t*-test. Error bars show ±SD. (**E**,**F**) Flow-cytometric analysis showing IAV host binding. The results are a representation of three independent experiments. ** *p* < 0.002, calculated by student’s *t*-test. Error bars show ±SD. (**G**) Isolation of lipid rafts as detergent-resistant membranes (DRM) and detection of IAV protein (M1). Actin served as internal control.

Since we observed that IAV co-localizes and interacts with host rafts during binding, we next investigated the effect of raft disruption on IAV binding. To achieve this, MBCD was used to deplete membrane cholesterol and disrupt the rafts (Figure 2A). After MBCD treatment, cell viability was also measured via MTT assay, which showed no significant cytotoxicity (Figure 2B). Since, MBCD is known to affect virus infectivity [18], treated A549 cells were washed thrice with 1× PBS to withdraw MBCD. Then, these cells were incubated with IAV at 4 °C for binding. After incubation, cells were fixed, permeabilized, and surface attached viruses were stained with anti-NP-FITC antibody (Green) for visualization under confocal microscope (Leica DM6000 CFS). We observed a drastic decrease in IAV host binding in raft-disrupted cells when compared with untreated control cells (Figure 2C). Subsequently, reduction in host binding was calculated in terms of mean fluorescence intensity and is graphically represented in Figure 2D.

Further, flow cytometry was performed with IAV incubated (4 °C) A549 cells (both untreated/treated) and similar reduction in IAV host binding was found in raft disrupted cells (Figure 2E,F). Subsequently, we isolated lipid rafts as DRM from A549 cells (both untreated/treated) incubated with IAV at 4 °C and observed the presence of IAV proteins in isolated raft fractions (Figure 2G), further validating our previous observations of IAV co-localization with host lipid rafts (Figure 1I). We also found significant reduction in IAV host binding in MBCD treated cells, similar to the confocal and flow cytometry results. Collectively, these data clearly show the importance of host lipid rafts during IAV binding.

### 3.3. Exploitation of Raft-Dependent Endocytosis by IAV as an Alternative Route for Host Entry

**Figure 3:**
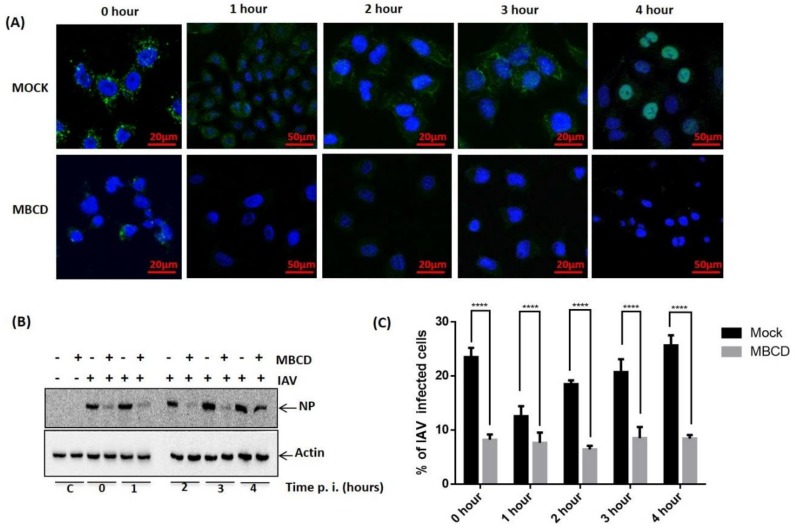

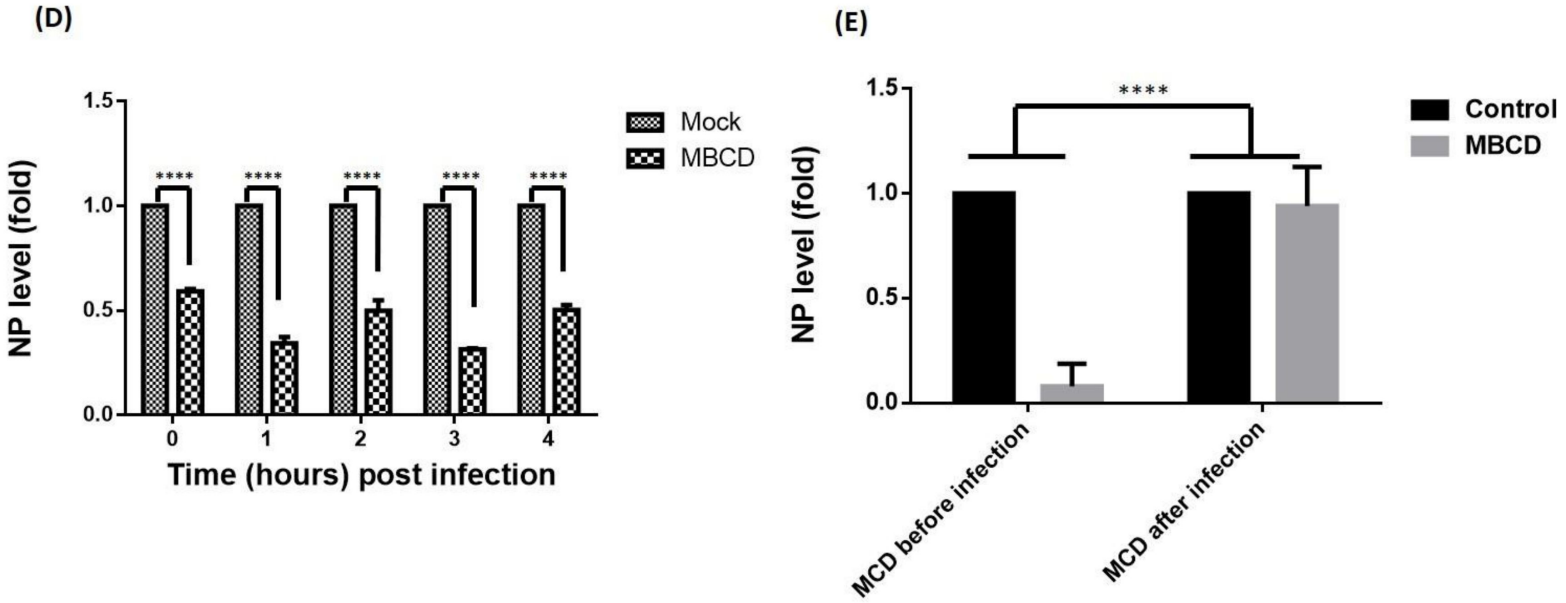
Exploitation of raft-dependent endocytosis by IAV for host internalization. (**A**) Confocal images of mock or MBCD treated, IAV infected (37 °C) A549 cells with indicated time of post IAV infection. Internalized IAVs were detected by staining with anti-NP-FITC antibody. (**B**) Analysis of IAV internalization via Western blot in mock or MBCD treated A549 cells showing drastic reduction in IAV entry in MBCD treated cells. Actin served as an internal control. (**C**) Flow cytometric analysis of IAV endocytosis in mock versus MBCD treated A549 cells. **** *p* < 0.0001, calculated by two way ANOVA. Error bars show ±SD. (**D**)**.** Relative quantitation of NP a central core of RNA genome (vRNA) by qRT-PCR. **** *p* < 0.0001, calculated by two way ANOVA. Error bars show ±SD. (**E**). Relative quantitation of NP vRNA in A549 cells that were MBCD treated before IAV infection and MBCD treated after IAV infection. **** *p* < 0.0001, calculated by two way ANOVA. Error bars show ±SD.

Since raft-dependent endocytosis is induced by multivalent ligand binding and reported to be cholesterol sensitive, we next sought to investigate if IAV utilizes raft-dependent endocytosis for host entry. To study this, A549 cells were either treated with MBCD to inhibit raft-dependent endocytosis [16] or left untreated (mock). Then these cells were washed with 1× PBS (thrice) to remove MBCD and incubated with IAV (1 hour) at 37 °C instead of 4 °C to allow endocytosis of surface attached IAVs. These cells (mock and treated) were subsequently incubated for different time intervals (0–4 h) post IAV infection to examine if raft disruption affected only IAV endocytosis or fusion of IAV membrane with the endosomal membrane was also affected by MBCD treatment. After the indicated time intervals, IAV infected cells (mock and MBCD treated) were harvested and analyzed for internalized IAV using anti-NP-FITC antibody. When visualized under confocal microscope, we observed significant and drastic reduction in IAV internalization in cells where raft-dependent endocytosis was disrupted (Figure 3A). Also, we observed IAV genome (detected via anti-NP-FITC antibody) present within the nucleus of host cell at 4-hour post IAV infection (Figure 3A, upper right panel). Further, when mock or MBCD treated, IAV infected (0–4 h) A549 cells were lysed and subjected to SDS-PAGE and Western blotting, a similar drastic reduction in IAV internalization was observed in MBCD treated A549 cells as evidenced with a reduced NP level (Figure 3B). Actin served as an internal control. However, detection of IAV NP in MBCD treated A549 cells, harvested at 4-hour post IAV infection, clearly indicates that MBCD treatment only inhibited raft-dependent IAV host entry and showed no effect on the fusion of viral membrane with endosomal membrane. Therefore, IAV genome was successfully released via membrane fusion event for viral replication. Next, flow cytometric analysis of mock and MBCD treated, IAV infected (0–4 h) A549 cells also showed similar significant reduction in IAV host entry in cells, where raft-dependent endocytosis was disrupted using MBCD (Figure 3C). Since IAVs have segmented RNA genome, therefore to further investigate IAV host entry via raft-dependent endocytosis and validate above observations, we subsequently isolated total cell RNA from mock and MBCD treated, IAV infected (0–4 h) A549 cells and converted to cDNA. Next, IAV host entry in mock (control) versus raft-disrupted cells was analyzed by qRT-PCR using primers against NP vRNA. We found similar reduction in IAV entry as evidenced by reduced NP vRNA level in cells with disrupted raft-dependent endocytosis (Figure 3D).

Further, to confirm the above hypothesis of IAV host entry via raft-dependent endocytosis, we either disrupted raft-mediated endocytosis in A549 cells before IAV infection or two-hours post-IAV infection. Next, these infected cells were incubated for 8 h at 37 °C, harvested and then subjected to total cell RNA isolation, converted to cDNA, and subsequently IAV entry was analyzed by qRT-PCR using NP primers. We found that while inhibition of raft-dependent endocytosis prior to IAV infection resulted in significantly reduced entry of IAV, raft disruption two-hour post IAV binding did not affected the IAV endocytosis (Figure 3E). Therefore, these data collectively demonstrate that IAV exploit the raft-dependent endocytosis for host internalization and entry.

## 4. Discussion

Here in the present study, we report that IAV select host lipid rafts during binding to the host cell to achieve high avidity. Additionally, we also demonstrate that IAV utilizes cholesterol sensitive raft-dependent endocytosis for host internalization and entry in addition to previously described endocytic routes for IAV [12,13].

Viruses very often exploit abundant but low affinity cell surface receptors for host attachment followed by binding with high affinity receptors and initiation of viral entry [19]. The IAV HA protein binds to host cell surface receptors’ sialic acid (SIA) for attachment and have weak affinity towards SIA molecules [11]. Consequently, IAV utilize multiple HA-SIA interactions to strengthen the host binding by achieving high avidity at the host cell membrane. Further, the plasma membrane (PM) is organized into sub-domains called “lipid rafts” that are enriched in glycoproteins and glycolipids and thus exhibit SIA clusters on the host surface [20]. These SIA clusters present in lipid rafts of host PM could offer a potential site for polyvalent interactions during IAV host binding. Takeda et al. had reported that newly translated HA protein is concentrated and aggregated in the lipid bilayer during viral assembly and budding, and this aggregation of HA molecules in the viral envelope is necessary for efficient viral host cell attachment and fusion during the subsequent infection [21]. We further demonstrate here that IAV is co-localized with host lipid rafts during host binding, and IAV proteins are detected in isolated lipid raft fraction when biochemically separated as DRM. Moreover, disruption of host lipid rafts via cholesterol extraction showed significant reduction in IAV host binding. Hence, we believe that these concentrated HA molecules in the viral envelope efficiently interact with host lipid raft’s SIA clusters, thus achieving high avidity on host PM. Extraction of PM cholesterol disrupts the rafts and hence leads to loss of SIA clusters, making it difficult for IAV to establish polyvalent interactions, resulting in drastically reduced IAV host binding. Therefore, we propose that host rafts and their SIA clusters are selected by IAV as host attachment factors for multivalent binding on the host surface.

Raft-dependent endocytosis induced by multivalent ligand binding is a well characterized route for internalization of ligands [22,23]. Since we report here that IAV select host lipid rafts for multivalent binding, it is reasonable that IAV might utilize the endocytic pathways induced and regulated by these micro-domains for host entry. During our experiments, the observation of reduction in IAV endocytosis in raft disrupted cells specify the utilization of raft-dependent endocytic routes for IAV entry. Also, the fact that no significant reduction in IAV internalization in cells where raft mediated endocytosis was disrupted two-hours post-IAV binding further strengthens our hypothesis. However, detection of IAV NP 4-hour post-IAV binding in cholesterol-depleted cells further indicates that there is no inhibition of pH dependent fusion of the viral-endosomal membrane leading to release of IAV genome for viral replication.

Whittaker et al. had used a similar approach and demonstrated that IAV may infect cells with disrupted caveolar uptake achieved by dominant-negative caveolin-1 and sterol binding drugs [24]. Though IAV infection in cells with dominant-negative caveolin-1 clearly indicates that IAV does not exploit caveolin-1 dependent caveolar endocytosis, use of sterol binding drugs during IAV binding and subsequent incubation may fail to remove cholesterol during IAV binding, showing lipid rafts or DRM independent IAV infection. However, for lipid raft disruption and inhibition of raft mediated endocytosis, we treated A549 cells with MBCD for 1 h prior to IAV infection to ensure cholesterol depletion, and then incubated them with IAV in the absence of MBCD due to the reported effect of MBCD on the viral envelope and infectivity [18], which showed significant reduction in both IAV host binding and entry. Also, exposure of cells to MBCD two-hour post IAV binding showed no such reduction. Therefore, we propose that IAV uses raft-dependent endocytosis for host entry as an alternative route in addition to the other previously known endocytic routes.

Viruses are known to utilize multiple endocytic routes to enter the host cell [25]. Although three different routes of raft-dependent endocytosis are suggested that share cholesterol sensitivity, they differ in their mechanisms. Caveolin-1 is reported to be crucial for caveolar endocytosis whereas non-caveolar dynamin-independent/dependent endocytoses are independent of caveolin-1 [16]. Further, SV40 virus is shown to be internalized via caveolae [26] as well as via a non-caveolar dynamin-independent route [27,28]. Similarly, endocytosis of CTB is reported to use both non-caveolar dynamin-independent/dependent routes [29,30]. The observed IAV infection in the presence of dominant negative caveoli-1 suggests that caveolar endocytosis may not be utilized for IAV internalization [24], and the other two known non-caveolar routes, i.e., dynamin dependent or dynamin independent, cannot be dissected here using MBCD because both are cholesterol sensitive. The co-localization of IAV with CTB suggests a similar route for endocytosis is exploited by IAV for host entry. Taken together, these data show that the IAV uses a host lipid raft as an attachment factor for polyvalent binding to the host surface and uses raft mediated endocytosis as an internalization route to gain entry inside the host cell.

Few host membrane proteins present in lipid rafts have been studied for their potential involvement in influenza virus entry. For example, C-type lectins have been suggested to be involved in influenza virus attachment to host cell. Further, dynamin proteins, epidermal growth factor receptors, and activated c-Met kinases have been reported to be critical for influenza virus internalization [12]. There are many questions that still need to be investigated. Does IAV follow dynamin-dependent or dynamin-independent routes or both routes during endocytosis? What are the IAV proteins that may interact with different proteins of raft-dependent endocytic pathways for entry? What is the molecular mechanism of this mode of IAV entry? The role of host surface proteins in IAV raft endocytosis needs to be explored. The answers of these questions will further clarify our understanding of IAV endocytosis.

## 5. Conclusions

Host lipid rafts has been reported to be critically involved in apical targeting, assembly, and budding of IAV, however, their role still remains elusive in the case of IAV binding and entry via endocytosis. In this study, we report that the IAV selects host lipid rafts for polyvalent binding to achieve high avidity on the host cell surface. Additionally, we also identify and demonstrate host lipid raft induced endocytosis known as “raft-dependent endocytosis” as an alternative internalization route for IAV in addition to the previous reported routes for IAV entry.

## References

[1] Taubenberger J.K., Morens D.M. (2008). The pathology of influenza virus infections. Annu. Rev. Pathol..

[2] Bouvier N.M., Palese P. (2008). The biology of influenza viruses. Vaccine.

[3] Simons K., Sampaio J.L. (2011). Membrane organization and lipid rafts. Cold Spring Harb. Perspect. Biol..

[4] Simons K., Ikonen E. (1997). Functional rafts in cell membranes. Nature.

[5] Chazal N., Gerlier D. (2003). Virus entry, assembly, budding, and membrane rafts. Microbiol. Mol. Biol. Rev..

[6] Takahashi T., Suzuki T. (2011). Function of membrane rafts in viral lifecycles and host cellular response. Biochem. Res. Int..

[7] Eierhoff T., Hrincius E.R., Rescher U., Ludwig S., Ehrhardt C. (2010). The epidermal growth factor receptor (EGFR) promotes uptake of influenza a viruses (IAV) into host cells. PLoS Pathog..

[8] Zidovetzki R., Levitan I. (2007). Use of cyclodextrins to manipulate plasma membrane cholesterol content: Evidence, misconceptions and control strategies. Biochim. Biophys. Acta.

[9] Skehel J.J., Wiley D.C. (2000). Receptor binding and membrane fusion in virus entry: The influenza hemagglutinin. Annu. Rev. Biochem..

[10] Sieben C., Kappel C., Zhu R., Wozniak A., Rankl C., Hinterdorfer P., Grubmuller H., Herrmann A. (2012). Influenza virus binds its host cell using multiple dynamic interactions. Proc. Natl. Acad. Sci. USA.

[11] Sauter N.K., Bednarski M.D., Wurzburg B.A., Hanson J.E., Whitesides G.M., Skehel J.J., Wiley D.C. (1989). Hemagglutinins from two influenza virus variants bind to sialic acid derivatives with millimolar dissociation constants: A 500-mhz proton nuclear magnetic resonance study. Biochemistry.

[12] Edinger T.O., Pohl M.O., Stertz S. (2014). Entry of influenza a virus: Host factors and antiviral targets. J. Gen. Virol..

[13] Lakadamyali M., Rust M.J., Zhuang X. (2004). Endocytosis of influenza viruses. Microbes Infect..

[14] Parton R.G., Richards A.A. (2003). Lipid rafts and caveolae as portals for endocytosis: New insights and common mechanisms. Traffic.

[15] Ewers H., Helenius A. (2011). Lipid-mediated endocytosis. Cold Spring Harb. Perspect. Biol..

[16] Lajoie P., Nabi I.R. (2010). Lipid rafts, caveolae, and their endocytosis. Int. Rev. Cell Mol. Biol..

[17] Chang T.H., Segovia J., Sabbah A., Mgbemena V., Bose S. (2012). Cholesterol-rich lipid rafts are required for release of infectious human respiratory syncytial virus particles. Virology.

[18] Sun X., Whittaker G.R. (2003). Role for influenza virus envelope cholesterol in virus entry and infection. J. Virol..

[19] Grove J., Marsh M. (2011). The cell biology of receptor-mediated virus entry. J. Cell Biol..

[20] Cohen M., Varki A. (2010). The sialome—Far more than the sum of its parts. Omics J. Integr. Biol..

[21] Takeda M., Leser G.P., Russell C.J., Lamb R.A. (2003). Influenza virus hemagglutinin concentrates in lipid raft microdomains for efficient viral fusion. Proc. Natl. Acad. Sci. USA.

[22] Nabi I.R., Le P.U. (2003). Caveolae/raft-dependent endocytosis. J. Cell Biol..

[23] Pelkmans L. (2005). Secrets of caveolae- and lipid raft-mediated endocytosis revealed by mammalian viruses. Biochim. Biophys. Acta.

[24] Sieczkarski S.B., Whittaker G.R. (2002). Influenza virus can enter and infect cells in the absence of clathrin-mediated endocytosis. J. Virol..

[25] Cossart P., Helenius A. (2014). Endocytosis of viruses and bacteria. Cold Spring Harb. Perspect. Biol..

[26] Pelkmans L., Kartenbeck J., Helenius A. (2001). Caveolar endocytosis of simian virus 40 reveals a new two-step vesicular-transport pathway to the ER. Nat. Cell Biol..

[27] Ewers H., Romer W., Smith A.E., Bacia K., Dmitrieff S., Chai W., Mancini R., Kartenbeck J., Chambon V., Berland L. (2010). Gm1 structure determines sv40-induced membrane invagination and infection. Nat. Cell Biol..

[28] Damm E.M., Pelkmans L., Kartenbeck J., Mezzacasa A., Kurzchalia T., Helenius A. (2005). Clathrin- and caveolin-1-independent endocytosis: Entry of simian virus 40 into cells devoid of caveolae. J. Cell Biol..

[29] Kirkham M., Fujita A., Chadda R., Nixon S.J., Kurzchalia T.V., Sharma D.K., Pagano R.E., Hancock J.F., Mayor S., Parton R.G. (2005). Ultrastructural identification of uncoated caveolin-independent early endocytic vehicles. J. Cell Biol..

[30] Lajoie P., Kojic L.D., Nim S., Li L., Dennis J.W., Nabi I.R. (2009). Caveolin-1 regulation of dynamin-dependent, raft-mediated endocytosis of cholera toxin-b sub-unit occurs independently of caveolae. J. Cell. Mol. Med..

